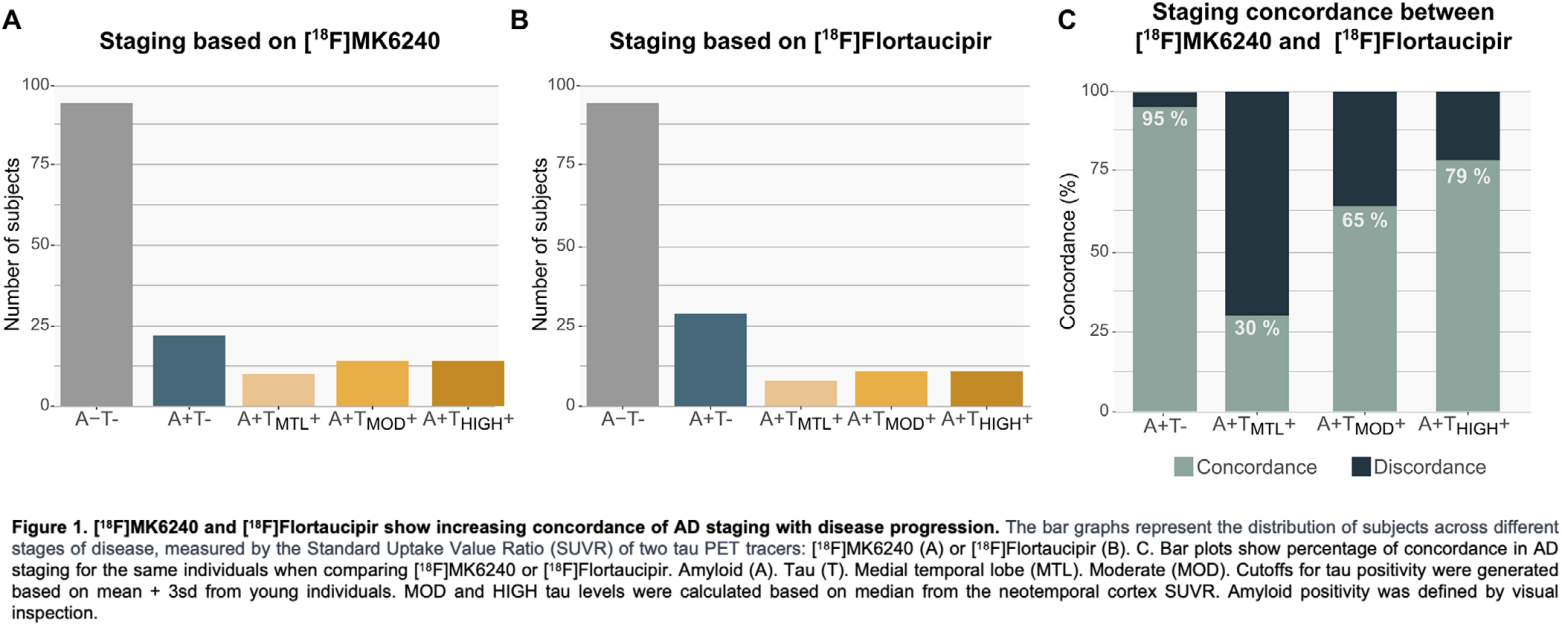# Alzheimer's disease staging with [^18^F]MK6240 and [^18^F]Flortaucipir ‐ the HEAD study

**DOI:** 10.1002/alz.094006

**Published:** 2025-01-09

**Authors:** Carolina Soares, Guilherme Povala, Guilherme Bauer‐Negrini, Firoza Z Lussier, Pamela C.L. Ferreira, Bruna Bellaver, Livia Amaral, Hussein Zalzale, Marina Scop Madeiros, Juli Cehula, Madeleine Bloomquist, Markley Oliveira, Cynthia Felix, Matheus Scarpatto Rodrigues, Emma Patrice Ruppert, Sarah Abbas, Eduardo R. Zimmer, Belen Pascual, Brian A. Gordon, Val J. Lowe, Hwamee Oh, David N. Soleimani‐Meigooni, Pedro Rosa‐Neto, Dana Tudorascu, William J. Jagust, William E Klunk, Suzanne L. Baker, Tharick A. Pascoal

**Affiliations:** ^1^ Universidade Federal do Rio Grande do Sul, Porto Alegre, Rio Grande do Sul Brazil; ^2^ University of Pittsburgh, Pittsburgh, PA USA; ^3^ Houston Methodist Research Institute, Houston, TX USA; ^4^ Washington University in St. Louis School of Medicine, St. Louis, MO USA; ^5^ Department of Radiology, Mayo Clinic, Rochester, MN USA; ^6^ Brown University, Providence, RI USA; ^7^ Memory and Aging Center, Weill Institute for Neurosciences, University of California, San Francisco, San Francisco, CA USA; ^8^ Translational Neuroimaging Laboratory, The McGill University Research Centre for Studies in Aging, Montréal, QC Canada; ^9^ Helen Wills Neuroscience Institute, University of California, Berkeley, Berkeley, CA USA; ^10^ Lawrence Berkeley National Laboratory, Berkeley, CA USA; ^11^ Department of Psychiatry and Neurology, Pittsburgh, PA USA

## Abstract

**Background:**

Utilizing PET amyloid‐beta (Aß) and tau for staging Alzheimer’s Disease (AD) has demonstrated potential in identifying individuals with varying degrees of disease severity, applicable to both clinical trials and practice. However, the diverse binding characteristics of tau tracers pose challenges to the application of this staging across different ligands. In this study, we evaluate a novel staging framework proposed by the AA working group, employing Aß PET and either [18F]MK6240 or [18F]Flortaucipir in individuals participating in a head‐to‐head study of tau PET tracers. This will provide insights into the robustness of this framework when applied across different tau ligands.

**Method:**

We evaluated 154 people (89 CU and 65 CI), with MK6240, Flortaucipir, Aß‐PET, MRI. SUVRs used inferior cerebellar gray as reference. Participants were categorized in Aß positivity(A), using visual read, and tau positivity (T) in medial temporal lobe(MTL) and neocortex(NEO). Tau MTL(TMTL) and NEO positivity were defined as average +3 SD of youngs. NEO moderate(TMOD) and high(THIGH) was defined as below and above median SUVR of NEO‐positive individuals. Individuals were grouped as: A‐T‐, A+T‐, A+TMTL+(early), A+TMOD+(intermediary), and A+THIGH+(advanced).

**Results:**

We found that the distribution of the number of individuals across the five AD stages was comparable when using both MK6240 and Flortaucipir tracers (Figure 1). However, upon examining the concordance within each group, we found a low concordance of 30% (3/10) between tracers in the early tau stage (A+TMTL+). Concordance increased in the intermediary stage A+TMOD+, with a rate of 65% (9/14), and further increased to 79% (11/14) in the advanced stage (A+THIGH+). The A+T‐ group exhibited the highest concordance between tracer results, at 95% (21/22).

**Conclusion:**

Our findings indicate that the application of this framework across different populations using different tau tracers can lead to a compatible distribution among groups. However, in a direct comparison, we noted that this did not necessarily result in high concordance between groups, as group shifts were observed that depended on the cutoffs used (data not shown). These results emphasize the necessity for harmonization of tau PET tracers prior to their utilization for stage determination.